# Application of Hollow Fibre-Liquid Phase Microextraction Technique for Isolation and Pre-Concentration of Pharmaceuticals in Water

**DOI:** 10.3390/membranes10110311

**Published:** 2020-10-29

**Authors:** Lawrence Mzukisi Madikizela, Vusumzi Emmanuel Pakade, Somandla Ncube, Hlanganani Tutu, Luke Chimuka

**Affiliations:** 1 Department of Chemistry, Durban University of Technology, P O Box 1334, Durban 4000, South Africa; 2 Department of Chemistry, Vaal University of Technology, Private Bag X 021, Vanderbjlpark 1900, South Africa; vusumzip@vut.ac.za; 3 Department of Chemistry, University of South Africa, Private Bag X6, Florida 1710, South Africa; ncubes@unisa.ac.za; 4 Molecular Sciences Institute, School of Chemistry, University of the Witwatersrand, Private Bag X3, Johannesburg 2050, South Africa; hlanganani.tutu@wits.ac.za (H.T.); luke.chimuka@wits.ac.za (L.C.)

**Keywords:** pharmaceuticals, water, hollow fibre-liquid phase microextraction, analytical methods

## Abstract

In this article, a comprehensive review of applications of the hollow fibre-liquid phase microextraction (HF-LPME) for the isolation and pre-concentration of pharmaceuticals in water samples is presented. HF-LPME is simple, affordable, selective, and sensitive with high enrichment factors of up to 27,000-fold reported for pharmaceutical analysis. Both configurations (two- and three-phase extraction systems) of HF-LPME have been applied in the extraction of pharmaceuticals from water, with the three-phase system being more prominent. When compared to most common sample preparation techniques such as solid phase extraction, HF-LPME is a greener analytical chemistry process due to reduced solvent consumption, miniaturization, and the ability to automate. However, the automation comes at an added cost related to instrumental set-up, but a reduced cost is associated with lower reagent consumption as well as shortened overall workload and time. Currently, many researchers are investigating ionic liquids and deep eutectic solvents as environmentally friendly chemicals that could lead to full classification of HF-LPME as a green analytical procedure.

## 1. Introduction

Pharmaceuticals are largely diluted in the environmental waters, hence their environmental concentrations are often found to range from low ng L^−1^ to µg L^−1^ levels [1,2]. The presence of pharmaceuticals in surface water sources is of environmental and health concern to both humans and aquatic life [3,4,5]. Due to the low environmental levels of pharmaceuticals and the complexity of the sample matrix, analytical equipment such as chromatography is unable to directly measure the concentration of these water pollutants. Therefore, sample preparation is a crucial step in the environmental monitoring of pharmaceuticals as it enables both analyte isolation and pre-concentration. Two conventional sample preparation techniques reported in the literature are based on liquid-liquid extraction (LLE) [6] and solid-phase extraction (SPE) [7]. Despite being successfully applied in the environmental monitoring of pharmaceuticals, both these sample preparation techniques have some drawbacks. The traditional LLE is known for its usage of high volumes of organic solvents which at times are toxic and hazardous to the environment as well as the operator [8]. Although SPE uses small amounts of organic solvents, it is a laborious and time-consuming process. Also, the traditional SPE sorbents often lack selectivity which results in co-extraction of humic substances [9]. Taking into consideration the necessity to adhere to green chemistry principles and to generate scientific data rapidly, there has been a growing interest in the development of miniaturized analytical methods that are reliable, fast, selective, and sensitive. In the last two decades, hollow fibre-liquid phase microextraction (HF-LPME) has been a promising tool for the efficient extraction and pre-concentration of pharmaceuticals from environmental waters prior to their chromatographic analysis. Furthermore, HF-LPME is a low cost efficient sample clean-up technique with pronounced selectivity [10].

Conventionally, HF-LPME involves transfer of analytes from a sample solution (the donor phase) where they exist in their uncharged state across a supported liquid membrane (SLM) into an acceptor phase inside the lumen of the hollow fibre. The SLM consists of a water-immiscible organic solvent embedded in the pores of a hollow fibre and acts as a clean-up barrier between the donor phase and the acceptor phase to avoid mixing of the two phases [11]. In a two phase extraction system, the water-immiscible organic solvent is used as both the SLM and the acceptor phase [12,13,14,15]. Whereas, an aqueous acceptor solution adjusted to adequate pH, depending on the acidic properties of the analytes, is common for the three phase extraction system [11,16,17,18,19]. Notably, the acceptor phase is also immiscible with the SLM. Both these extraction systems have been used in the extraction of pharmaceuticals from water samples (Table 1).

Having been introduced for the first time in 1999 [20], HF-LPME has now been known for over 20 years. However, there are few review articles reported in the literature that have critically evaluated the analytical applications of this important sample preparation technique [21,22,23]. To summarize, Bello-Lopez et al., 2012 reviewed all analytical applications for HF-LPME reported in the literature [21], while Han and Row, 2012 only focussed on applications to environmental and biological samples [22]. Other review articles gave the overview of the LPME technique with great focus directed towards its extraction principles, historical developments. and performance [24,25,26,27,28,29,30,31,32]. Due to the initiative to develop and utilize the green analytical procedures in chemical analysis, LPME fulfils the requirements for its classification as a green analytical procedure [30]. This is mainly due to the low consumption of organic solvents in its operation. In a review article, Kokosa, 2019 discussed the solvent selection recommendations for LPME in an attempt to ensure the complete greenness of this technique by avoiding the usage of trace amounts of toxic organic chemicals [30]. In a different perspective, a recent review article presented by Khan et al., 2020 focussed on the applications of HF-LPME technique followed by the analytical instrumental quantitative analysis for heavy metal ions and pharmaceuticals [23]. The present review article focusses entirely on discussing the applications of HF-LPME in the quantitative analysis of pharmaceuticals in water. The performance of HF-LPME is presented and the modifications of the technique to enhance its ability to isolate and pre-concentrate pharmaceuticals in water are discussed.

## 2. Experimental Set-up, Modes, and Theoretical Principles

### 2.1. Modes, Principles, and Theory of HF-LPME

**Figure 1:**
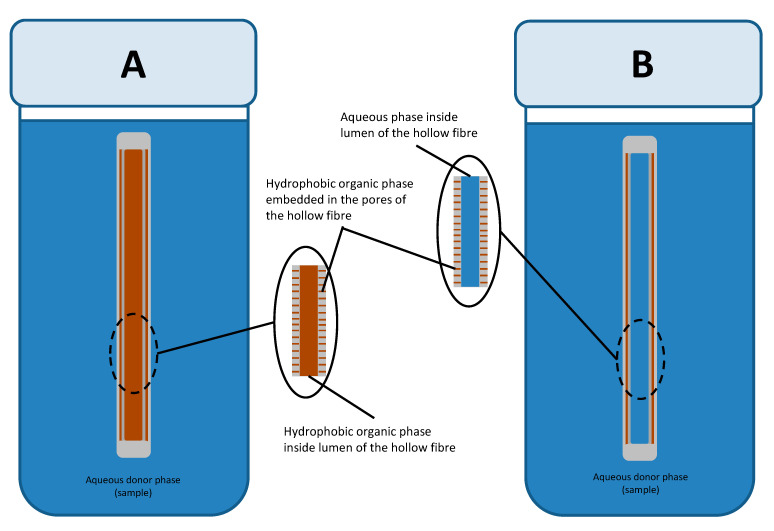
Illustrations of the two phase (**A**) and three phase HF-LPME (**B**).

There are two modes of operation for HF-LPME based on the number of phases involved [32]. In this regard, there is the two-phase HF-LPME that utilizes two phases and the three-phase HF-LPME which utilizes three phases (Figure 1). In the two-phase HF-LPME (Figure 1A), a lipophilic organic phase is impregnated into the pores of the HF and acts as the SLM. The same organic phase is filled into the lumen of the HF and acts as the acceptor phase [33]. In a three-phase HF-LPME mode (Figure 1B), the lipophilic organic phase is only impregnated into the pores of the hollow fibre while the lumen is filled with an aqueous acceptor phase. Some three phase HF-LPME studies have reported an organic acceptor phase immiscible with the organic SLM phase. For example, n-dodecane has been used as an SLM phase with an alcohol (methanol) [19] or an alkane (n-undecane) [34] as the acceptor phases.

Applications of the two-phase HF-LPME are mainly for analytes with high octanol–water coefficient (Kow) values [35]. Transfer of the analytes occurs by partitioning from the aqueous donor phase into the organic SLM and their eventual diffusion into the same organic phase in the lumen of the HF [36]. The organic acceptor phase is preferentially injected directly into a GC instrument or else it needs to be reconstituted if an LC system is used for analysis. The ratio of the analyte concentrations in the organic acceptor phase to the concentration in the aqueous donor phase when the system reaches equilibrium (Equation (1)), is known as the distribution constant (K) and can be represented as Equation (2). The amount of analyte transferred is calculated as the recovery (R) or extraction efficiency (E) using Equation (3). Good recoveries are obtained for analytes with high K values.

(1) $$C_{aq.dp}↔C_{org.ap}$$

(2) $$K=C_{org.ap}^{eq}/C_{aq.dp}^{eq}$$

(3) $$R=K.V_{org.ap}/\left(K.V_{org.ap}\times V_{aq.dp}\right)\times 100$$

where $C_{aq.dp}$ is the analyte concentration in the aqueous donor phase, $C_{org.ap}$ is the analyte concentration in the organic acceptor phase. K is the distribution constant, $C_{org.ap}^{eq}$ is the analyte concentration in the organic acceptor phase, and $C_{aq.dp}^{eq}$ the analyte concentration in the aqueous donor phase, when the system has reached equilibrium. R is analyte recovery, $V_{org.ap}$ and $V_{aq.dp}$ are the volumes of the organic acceptor phase and aqueous donor phase, respectively.

In a three-phase HF-LPME mode, transfer of the analytes occurs by partitioning from the aqueous donor across the organic SLM into the aqueous acceptor phase in the lumen of the hollow fibre (Equations (4)–(6)). The distribution coefficient (K) is therefore the product of distribution coefficients across the three phases (Equation (7)). The amount of analyte transferred from the aqueous donor phase into the aqueous acceptor phase is then calculated using Equation (8).

(4) $$C_{aq.dp}↔C_{org.SLM}↔C_{aq.ap}$$

(5) $$K_{SLM/dp}=C_{org.SLM}^{eq}/C_{aq.dp}^{eq}$$

(6) $$K_{ap/SLM}=C_{aq.ap}^{eq}/C_{org.SLM}^{eq}$$

(7) $$K=K_{SLM/dp}.K_{ap/SLM}$$

(8) $$R=(K_{SLM/dp}K_{ap/SLM}V_{ap})/\left(K_{SLM/dp}K_{ap/SLM}V_{ap}+K_{SLM/dp}V_{SLM}+V_{dp}\right)\times 100\;$$

where $C_{org.SLM}$ is the analyte concentration in the organic SLM and $C_{aq.ap}$ is the analyte concentration in the aqueous acceptor phase. $K_{SLM/dp}$ is the analyte distribution coefficient between the organic SLM and the aqueous donor phase, $C_{org.SLM}^{eq}$ is the analyte concentration in the organic SLM phase and $C_{aq.dp}^{eq}$ is the analyte concentration in the aqueous donor phase, when the system has reached equilibrium. In Equation (6), $K_{ap/SLM}$ represents the analyte distribution coefficient between the aqueous acceptor phase and the organic SLM phase, $C_{aq.ap}^{eq}$ is the analyte concentration in the aqueous acceptor phase, when the system has reached equilibrium. K is the distribution coefficient from the aqueous donor phase across the organic SLM into the aqueous acceptor phase. R is the analyte recovery, $V_{ap}$ the volume of the acceptor phase, $V_{dp}$ is the volume of the donor phase.

Applications of three phase HF-LPME are mainly in extraction of acidic or basic analytes with ionizable groups [33]. The idea is to keep the analytes neutral in the aqueous donor phase and charged in the aqueous acceptor phase. For acids, the pH of the donor phase is kept below the pKa value of the analyte. The pH of the acceptor is kept above the pKa value so that the analytes that diffused across the SLM into the lumen remain ionized and are prevented from back-extraction. For basic analytes, the donor phase pH is kept above the pKa values so that they remain in their neutral form and the acceptor phase pH below the pKa value to keep them ionized.

The performance of an HF-LPME method is usually defined in terms of a pre-concentration factor (PF) or enrichment factor (E_f_) rather than extraction efficiency. For the calculation of extraction efficiencies (Equations (3) and (8)), there is the need to know precisely the volume of the acceptor solvent in the lumen of the HF. Our experiences with hollow fibres show that it is always difficult to recover and precisely determine the volume of the acceptor phase that was injected into the lumen of the hollow fibre. In this regard, a simple comparison of the initial analyte concentration in the donor phase before extraction and the final concentration in the acceptor phase after extraction is preferred. This is calculated as a ratio using Equation (9). Most HF-LPME applications in the literature therefore use enrichment factors (Equation (9)) to report its performance [37].

(9) $$E_{F}=C_{ap}^{eq}/C_{aq.dp}^{eq}$$

where $E_{F}$ is the enrichment factor, $C_{ap}^{eq}$ is the analyte concentration in the acceptor phase, and $C_{aq.dp}^{eq}$ is the analyte concentration in the aqueous donor phase, when the system has reached equilibrium.

### 2.2. Pros and Cons

The dimensions and design of the fibre make HF-LPME a favourable sample preparation technique in terms of efficiency as well as the amount of solvents used. For example, its wall thickness, pore size, and internal diameter are all in the micrometre scale. This, in addition to the reported lengths of the fibre employed during extraction ensures that micro-scale volumes of the organic SLM and the acceptor phases are used, making the HF-LPME a greener technique. The maximum acceptor phase volume reported in the analysis of pharmaceuticals in aqueous samples is 100 µL in which a 53.3 cm long hollow fibre was used [38]. Most of the studies on pharmaceuticals in aqueous samples have reported 8 cm long unsealed fibre with a total possible volume of 22.6 µL [11,16,39]. Elsewhere, some studies have reported lengths as short as 1.8 cm (<5 µL) for analysis of flunitrazepam in biological samples [40]. With the donor phase volume reported in the mL–L scale, the HF-LPME technique allows for high enrichments of the analytes from the donor to acceptor phase with Ho et al., 2007 achieving a record 27,000 in the extraction of antidepressants in wastewater [41]. High enrichment factors of ibuprofen in wastewater (>15,000) have also been reported [38].

In addition, the phases are immiscible yet in continuous contact with a lipophilic organic phase mechanically embedded on the fibre pores. This eliminates the formation of emulsions while at the same time providing a continuous, real-time design with a potential for automation and on-line hyphenation with analytical instrumentation [21,28]. Automation of the HF-LPME for analysis of pharmaceuticals has already been reported in the literature [19]. While automation brings added costs related to the instrumental part, the costs are greatly reduced in relation to reagents consumption, shortened overall workload, and time, and fewer personnel need to be paid as the instrument does the work instead [42]. The SLM is also mechanically stable and the experimental set-up can be stirred to enhance the extraction process. The HF-LPME is a versatile technique with applications in the analysis of both organic and inorganic analytes of varying polarities with successful applications in environmental, food and biomedical analysis [21,27,36,43].

Unfortunately, the SLM is not devoid of limitations notably those related to the kinetics of mass transfer, stability of the SLM, and the charge of the analytes. Notably, the HF-LPME is only suitable for analytes with functional groups that ionize over a particular pH range. Mass transfer is generally slower, and the experimental set-up needs to be stirred while a carrier molecule is needed to facilitate transfer of very polar analytes [23,44]. Both parameters need to be optimized which further prolongs the application process. For example, stirring is known to increase transfer of analytes but when done excessively it will affect membrane stability. Furthermore, processes such as pressure difference, dissolution and evaporation of organic phase or dispersion into the adjacent phases (donor and acceptor) are known to cause the loss of the organic layer from the supported membrane, thus leading to membrane instability [45]. Membrane stability has been improved through strip dispersion and feed dispersion methods as well as the incorporation of ionic liquids as SLM phases as discussed in Section 5.

### 2.3. Carrier-Mediated HF-LPME

Partitioning of relatively polar hydrophilic analytes from an aqueous donor phase is usually very low. Such analytes include those that have both basic and acidic functional groups that ionize differently at the same pH level therefore the analytes exist in their charged state over a wide pH range as well as those with very low log K_ow_ values. Effective diffusion into the organic SLM is then achieved via carrier-mediated transfer using ion-pair reagents [46]. For a two-phase HF-LPME mode, a hydrophobic ion-pair reagent is added to the aqueous donor phase to pair with the target analytes. The polar groups of the reagent combine with the analyte functional groups forming a relatively neutral ion-pair complex that easily diffuses into the organic phase in the lumen of the HF. Applications of ion-pair two phase HF-LPME in analysis of pharmaceuticals in aqueous samples is summarized in Table 1.

In a three-phase HF-LPME mode, a carrier molecule is dissolved into the SLM [46]. The carrier molecule must be lipophilic so that it remains in the organic SLM. It binds with the charged analyte at the donor phase-SLM interface forming a neutral hydrophobic ion-pair complex that diffuses into the organic SLM phase. At the SLM-acceptor phase interface, the carrier molecule releases the analyte into the aqueous acceptor phase and picks up some counter ion to maintain electro neutrality within the SLM. The counter ion is itself released into the donor phase at the donor phase-SLM interface as the carrier picks up another analyte. To prevent analyte back-extraction, the pH of the acceptor phase is adjusted to levels that would maintain a high concentration of counter ions [21]. The driving force is therefore maintenance of a high counter ion concentration gradient between the acceptor and donor phases. For extraction of basic analytes, H^+^ ions are used as counter ions and therefore, the acceptor phase pH is kept low (high H^+^ ions) while a high pH level (high OH^-^ counter ions) is essential for extraction of acidic ions. Some halides such as Br^-^ and Cl^-^ (NaBr and NaCl acceptor phases) have been reported as counter ions for acidic pharmaceuticals [47].

The choice of a carrier molecule depends on the functional group(s) of the analyte [21]. For acidic analytes, cationic ammonium-based salts such as tetrabutylammonium (TBA), *N*-methyl-*N*,*N*,*N*-trioctylammonium chloride (Aliquat 336) are used as carriers while basic analytes are ion-paired with anionic carriers especially bis(2-ethylhexyl) hydrogen phosphate (DEHPA) and tri-n-octylphosphine oxide (TOPO). Those applied in analysis of pharmaceuticals in environmental samples are discussed in Section 3.1.

### 2.4. HF-LPME Experimental Set-up

**Figure 2:**
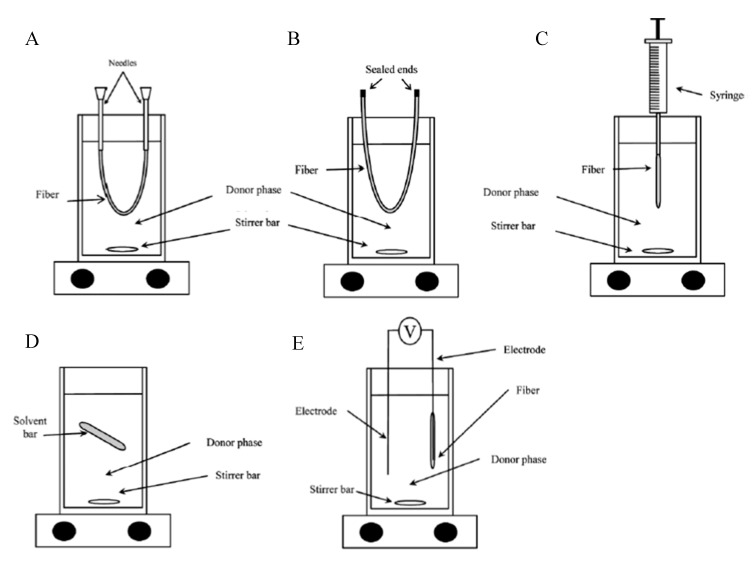
Typical HF-LPME experimental set-ups: (**A**) needles inserted at both ends of the hollow fibre, (**B**) ends of the hollow fibre both sealed; (**C**) one hollow fibre end sealed, other end held by a chromatographic syringe; (**D**) free stirring fibre with two ends sealed; (**E**) sealed hollow fibre connected to a DC-supply. Adopted from Bello-López et al., 2012 [21].

The most common hollow fibre used in both HF-LPME modes is the Q3/2 Accurel polypropylene supplied by Membrana GmbH, Wuppertal, Germany [48]. The Q3/2 fibre has a pore size of 0.2 µm and 70% wall porosity. Its inner diameter and wall thickness are 600 µm and 200 µm, respectively. However, other fibre types with smaller dimensions such as the plasmaphan polypropylene hollow fibre (330 µm inner diameter, 140 wall thickness, 0.4 µm pore size) have been reported for analysis of antidepressant drugs in wastewater [41]. Preparation of the fibre involves cutting the fibre into a specific length. Depending on the set-up, the fibre can be heat-sealed at one end and then filled with the acceptor phase. The other end can also be heat-sealed (Figure 2B) or held with a chromatographic syringe or wire (Figure 2C). Alternatively, one end can be stoppered with a needle followed by filling the lumen with the acceptor phase and finally stoppering the other end with another needle (Figure 2A).

When filling the lumen of the fibre, studies have reported that the acceptor phase is pushed in until bubbles appear on the outer surface of the fibre. The other end of the fibre is heat-sealed or held with a wire/syringe with a diameter that compliments the internal diameter of the fibre. The bubbles on the walls of the fibre are wiped with a paper towel. For a three-phase HF-LPME in which an aqueous solution is used as the acceptor phase, the fibre is first dipped in an organic solvent to be used as the SLM for a few seconds with most studies reporting ≤15 s. This allows the lipophilic organic phase to embed on the pores of the fibre. Excess organic phase loosely attached on the surface of the HF is removed by dipping the fibre in deionized water for a few seconds typically ≤5 s. The fibre containing the acceptor phase with its pores impregnated with an organic phase is finally placed in the aqueous donor phase to allow transfer of the analytes. When extraction has reached equilibrium, the fibre is removed, and a chromatographic syringe used to draw the acceptor phase from the lumen of the fibre. Effort is made to ensure all the acceptor phase is drawn into the syringe so that the true volume can be recorded. The acceptor phase is then diluted to ensure its pH is within the working range of the column and tubing of the chromatographic instrument. The diluted acceptor phase is finally injected into the instrument for analysis.

Typical experimental setups of conventional HF-LPME are shown in Figure 2A–C. Most studies have used a wire in place of the chromatographic syringe in Figure 2C depending on availability of syringes and the number of experiments set up for extraction. Figure 2D,E represent set-ups for advanced forms of HF-LPME. Figure 2E represents an electro-membrane (EME) in which an electric field is used to enhance transfer of analytes in a three-phase HF-LPME set-up. This was first demonstrated in 2006 by Pedersen-Bjergaard and Rasmussen using two electrodes: one inserted in the donor phase and the other in the acceptor phase [49]. Effective electrokinetic migration is achieved by adjusting the pH of the donor and acceptor phases to keep the analytes in their ionized forms in both phases [21]. The negative electrode is inserted in the donor phase for analysis of acidic analytes and the set-up is reversed for analysis of basic analytes where the negative electrode is inserted into the acceptor phase. Figure 2D represents solvent bar microextraction (SBME). In this set-up, the hollow fibre is sealed at both ends and submerged in the aqueous sample solution [32]. During stirring, the fibre (and the acceptor phase) freely tumbles inside the aqueous solution facilitating the transfer of analytes across the SLM. Advances in this technique include inserting a steel wire to enable magnetic stirring (Guan 2017).

## 3. Critical Parameters Affecting the Extraction Process

Various factors such as extraction solvent, extraction volume, extraction time, stirring rate, temperature, and ionic strength of the sample solution play a crucial role in the performance of the HF-LPME process [22,28,50]. These factors have been reported to affect the LPME efficiency, therefore, they have to be optimized in order to achieve the maximum extraction of analytes from the sample matrix. With considerations that the influence of these factors on LPME for a wide range of analytes have been reviewed previously [22,28], herein, we provide a critical review on how each factor has been reported to affect the extraction of pharmaceuticals only from water samples.

The optimization process is usually done by applying the one-factor-at-a-time (OFAT) approach. In this approach, the operator systematically changes one extraction parameter at a time while keeping all other variables constant. OFAT assumes that the optimum effect of a factor is the same in the entire range of another effect. An alternative to this approach is a multivariate optimization process where several parameters and their interactive effects are optimized simultaneously. The approach considers the following points; (1) the impact of some factors is more pronounced than others, (2) optimum performance is also affected by interactive effects of critical parameters and, (3) the optimum effect of a single factor cannot be the same in the entire range of another effect. Various statistical software packages used in multivariate optimization are well described in the literature [12], however those mentioned in the HF-LPME for pharmaceuticals in aqueous samples include the Design-Expert (DOE software, Stat-Ease, Minneapolis, MN, USA) and Minitab 18 (State College, PA, USA) used in the studies focussing on the extraction of carbamazepine [12] and selected antiretroviral drugs [16], respectively. A strategy based on multivariate optimization helps in identifying critical parameters that have a significant effect on the extraction of the analytes among parameters that have been predicted to have a potential impact on the performance of the extraction technique. Such factors are further paired according to the extent of their impact and their interdependence (or interactive effects). For example, Mlunguza et al., 2020 used Minitab 17 to identify four critical factors (SLM carrier composition, extraction time, donor phase, and acceptor phase pH) from six possible factors with a potential to affect extraction of antiretroviral drugs from wastewater samples [16]. These were further paired according to their level of impact, and the optimum interactive responses predicted by fitting a second-order quadratic model. The authors reported that the optimum interactive effects of the four critical parameters helped them design a robust HF-LPME experimental set-up that gave the best analyte enrichments possible.

### 3.1. Supported Liquid Membrane

Choosing an appropriate organic solvent is crucial for the optimal extraction and enrichment performance of an HF-LPME technique especially for a two phase HF-LPME where the SLM organic solvent is used as the acceptor phase [14,28,51]. Organic solvents exhibiting high extraction efficiency (enrichment) for the targeted analytes should be used [52] but sometimes a compromise has to be made particularly when extracting a group of compounds with different polarities as some might have higher extraction affinity for a different solvent. The efficacy of the selected organic solvent is measured against its ability to extract analytes of interest efficiently and should be compatible with the fibre, immiscible with the donor solutions, and exhibit low volatility (high boiling point) to prevent volatile and diffusion losses during extraction [52,53]. Many studies have reported on the use of pristine hydrophobic organic solvents for the enrichment of pharmaceuticals from the aqueous phase with 1-octanol emerging as the popular choice in the analysis of pharmaceuticals [14,38,54,55,56,57]. For example, Zhang et al., 2013 investigated tributylphosphate, 1-octanol, and 1-butanol and found that extraction of non-steroidal anti-inflammatory drugs (NSAIDs) was optimum when 1-octanol was used [14]. In addition to possessing an active hydrogen atom that specifically interacts with the oxygen in carboxyl groups of targeted compounds, 1-octanol has lower volatility and larger viscosity, thus abating its loss to the solvents.

Some studies utilizing the three phase HF-LPME for pharmaceuticals especially in aqueous samples have also conducted optimization studies to select the best organic solvent for the SLM phase. There seems to be no agreement by researchers on the best SLM solvent for analysis of pharmaceuticals in aqueous environmental samples but 1-octanol and dihexyl ether appear prominently in most studies (Table 1). For example, the 1-octanol SLM gave better enrichments for sulfonamides and fluoroquinolones [56,58] as well as estrogens in wastewater [59] while dihexyl ether was the better SLM for fluoroquinolones [37] and antidepressants [41] as well as oestrogens in tap and sewage water [34]. However, in some studies using non-carrier three phase HF-LPME for pharmaceuticals in aqueous samples, dihexyl ether was used as the SLMs [11,55]. For carrier-mediated three phase HF-LPME, optimization of the SLM has mainly focused on the composition of the carrier molecule in the SLM phase. The choice of the SLM organic phase and the carrier molecule is usually based on recommendations of previous studies. The most common SLM organic solvent in these carrier-mediated three phase HF-LPME studies has been dihexyl ether [28]. In this regard, optimization of the composition of the carrier in the dihexyl ether SLM for the extraction of pharmaceuticals in water has been reported [16,39]. On the other hand, both the organic phase and the carrier composition have been optimized. For example, Shariati et al., 2009 found that octanol containing 10% (*w*/*v*) aliquat-336 was optimum for extraction of tetracycline antibiotics in tap water [44] while Yamini et al., 2006 reported an optimized 20% aliquat-336 in dihexyl ether in analysis of salbutamol and terbutaline in tap, well and river water [47]. Optimization of the carrier composition is essential because while an increase is expected to enhance transfer of analytes, an excess will affect the viscosity of the SLM. In this regard, most studies have noted an increase of enrichments with increase in carrier composition up to about 10–20% (*w*/*v*) (Table 1). Above these optimized conditions, there is always a decline in enrichments. However, some studies have optimized the organic phase but not the carrier composition such as the study by Msagati and Mamba, 2018 who used hexylamine + 5% TOPO for analysis of sulphonamides but only hexylamine was selected from two other organic solvents whereas the TOPO composition was not optimized [60].

### 3.2. Sample and Acceptor Phase pH

The HF-LPME was mainly designed for the isolation of ionic or polar analytes such as acids, bases and metals [61]. The charge of these analytes can be modified by the changes in solution pH. The diffusion of an analyte through the SLM containing an organic solution occurs when the analyte in the donor phase is present in its neutral form [50,61,62]. On the other side, the acceptor phase pH should be well adjusted so that the analytes become charged inside the lumen of the hollow fibre to avoid back extraction since only neutral species can diffuse through the membrane [62]. For example, Li et al., (2015) indicated that an increase in the sample pH reduced the solubility of their analytes (β-blockers) in water (sample solution) and enhanced the solubility in the organic solvents [13]. Hence, pH has been described as an important factor that needs to be well optimized for each analyte when conducting the HF-LPME [50].

A good understanding of the charge of each analyte at a given pH based on its pK_a_ value is critical in optimization of the aqueous solution pH. The pK_a_ value of an analyte may be used to estimate the pH range of the donor phase and the acceptor phase that can keep the analyte in a particular charged state. [54]. For example, to keep an acidic analyte neutral in the donor phase, the pH of the solution must be lower than its pKa value. Likewise, the acceptor pH must be higher than the pK_a_ value to keep the analyte in its ionized form. To demonstrate this, the optimum sample pH used by Es’haghi, 2009 when extracting NSAIDs (ibuprofen, naproxen, and ketoprofen) with pK_a_ values ranging from 4.15 to 5.2 was 3.5 to keep them neutral [54]. These acidic pharmaceuticals are ionized at higher pH values (pH > 4), which means the acceptor solution should be kept at neutral to basic pH. Elsewhere, studies have reported a donor phase pH of 3 during the extraction of various NSAIDs (pK_a_ ~ 4), with ammonium carbonate buffered at pH 9 [63] and pH 10 [39] utilized as the acceptor solution which allowed for the deprotonation of analytes leading to their isolation from water samples. Several research groups have shared the same sentiments for NSAIDs extraction where they utilized the donor phase pH and acceptor phase pH in the ranges of 1.5–2 and 9.5–12.5, respectively [11,14,55,62,64,65,66].

On the other hand, basic analytes are best extracted at acidic conditions (low donor phase pH) as they are known to ionize at alkaline pH [15,60]. In this context, Msagati and Mamba used a donor phase pH of 6.1 during the extraction of 17β-estradiol (pK_a_ = 10.46), estrone (pK_a_ = 10.34), and 17β-estriol (10.38) [60], while Zou et al., (2014) preferred pH 2 for isolation of 17β-estradiol, estrone, diethylstilbestrol (pK_a_ = 8.63) and bisphenol A (pK_a_ = 10.29) in water [15]. The optimized pH conditions for other analytes in different studies are summarized in Table 1. The presented observations imply that the simultaneous extraction is most appropriate for the compounds with similar properties such as pK_a_ values. In the extraction of antiretroviral drugs, our research group achieved a wide distribution of enrichment factors that ranged from 24 for efavirenz (pK_a_ = 12.52) to 111 for tenofovir disoproxil (pK_a_ = 18.59) [16]. In that study [16], we had to compromise as the enrichment factor for efavirenz increased when using high acceptor phase (HCl) concentration and SLM carrier composition, while these conditions negatively affected the enrichment of the other two analytes (tenofovir disoproxil and emtricitabine).

Notably, as observed elsewhere for the extraction of 4′-isobutylacetophenone and other transformation products of anti-inflammatory drugs in water and sludge [67], the pH did not affect the HF-LPME process significantly. The authors of that study accredited their results to the chemical structures and properties of the analytes.

### 3.3. Extraction Time

In HF-LPME, sufficient extraction time is required to ensure the complete migration of analytes from the sample solution to the acceptor phase. In theory, the longer extraction times lead to a faster partition equilibrium of analytes being reached between the sample solution and acceptor phase [14]. Thus, the mass transfer is a time dependent process as described by Equation (9) [68]. It is crucial to optimize the extraction time as this parameter is the key factor that impacts the duration of the analytical method. Based on Table 1, a complete extraction of pharmaceuticals from water can be achieved within 15 min. The duration of the extraction is likely to be influenced by the analytes and the acceptor solution. Long extractions lasting for more than an hour have also been reported in the literature [15,64,69].

### 3.4. Stirring Rate

It is crucial to agitate the sample solution during the HF-LPME as this facilitates the mass transfer process of analytes and reduces the time required to reach the equilibrium between the sample solution and the organic phase [50]. Higher stirring rates are known to speed-up the flow of analytes through the SLM [61]. However, too high a stirring speed can reduce the contact area between sample solution and organic solvent, and subsequently produce air bubbles on the surface of the hollow fibre [63]. Li et al., 2015 also suggested that excessive stirring could cause the loss of the organic solvent immobilized in SLM leading to the reduction of the extraction efficiency as well as the precision of the extraction method [13]. As a result, the stirring rate is one of the key parameters that is often optimized during HF-LPME. Stirring rates often used for HF-LPME of pharmaceuticals in water samples range from 100 to 1100 rpm (Table 1). The varying stirring rates are likely to be influenced by the sample volume and individual analytes.

### 3.5. Temperature

Although not optimized in numerous studies, temperature has been reported as a critical parameter in the extraction process due to its relation to the thermodynamics and kinetics of the extraction [13]. Furthermore, temperature is known to facilitate the mass transfer of the analytes from the sample (aqueous) solution to the organic phase [13,50]. Sufficient mass transfer for naproxen and nabumetone from water into a 1-undecanol acceptor phase was achieved at 45 °C [50]. In this case, the analytical signals for both pharmaceuticals increased from 25 to 45 °C, with no changes observed when the temperature was further increased to 55 °C [50]. In a different study, the extraction of β-blockers from environmental water samples was performed at 60 °C [13]. Above 60 °C, Li et al., 2015 observed a decline in the extraction efficiency due to higher temperatures producing high vapor pressure of the extraction solvent resulting in the vapor coming out from the top of the hollow fibre connected with a needle and dissolving back into the water samples [13]. In the same study, lower extraction temperatures resulted in inefficient mass transfer of analytes. However, Zhang et al., 2013 reported an optimum temperature of 20 °C for the extraction of NSAIDs in water samples into a 1-octanol acceptor phase with the authors observing a loss of the acceptor phase at higher temperatures which led to lower extraction efficiencies [14]. This means the usage of higher temperatures for the extraction should be applied in consideration of the nature of the utilized acceptor solution and organic solvent.

Notwithstanding its importance, the inclusion of heat in the extraction process performed at high temperatures requires the use of energy which is not encouraged in green analytical chemistry principles [70]. A compromise can be made in this regard by extending the extraction time in order to allow for sufficient mass transfer without increasing the temperature.

### 3.6. Ionic Strength

The addition of the salt into the sample solution increases the ionic strength which is likely to lessen the solubility of the organics in the aqueous phase through the salting out effect, thereby resulting in improved extraction efficiency [15,22,50,61]. In the HF-LPME of naproxen and nabumetone from aqueous solutions, the analyte responses increased when the potassium chloride concentration was varied from 0 to 4% (*w*/*v*) in aqueous samples [50]. Potassium chloride levels beyond 4% (*w*/*v*) did not cause any significant changes in analyte responses for both pharmaceuticals [50]. In a different study, the extraction efficiency for fluoroquinolones was increased due to the addition of 2 M sodium sulphate in the donor phase [56].

However, the ionic strength should be carefully optimized as high salt concentrations in the sample solution have the ability to alter the physical properties of the extraction film which causes a reduction in the diffusion rates of the target compounds into the organic phase [22,61]. In the HF-LPME of salicylic acid, the sodium chloride dissolved in the aqueous solution increased the ionic strength of the donor solution, causing a decrease in the analyte solubility [61]. However, the excessive addition of sodium chloride to the sample solution affected the analyte signal negatively, which was attributed to the increased ionic strength which has the potential to change the physical properties of Nernst thin film, leading to the reduction of the mass transport in the interface between donor solution and supported liquid membrane [61]. Also, the change in the ionic strength alters the viscosity of the donor phase and negatively affects the kinetics of the extraction process [63]. In this instance, during the HF-LPME of β-blockers from water samples, the addition of sodium chloride from 0 to 30% (*w*/*v*) into the sample solution was accompanied by a decrease in the extraction efficiencies [13]. Li et al., (2015) attributed these results to the increase of donor phase velocity which hindered the mass transfer due to the addition of sodium chloride [13].

Notably, it is also possible for the variations of the ionic strength not to influence the extraction efficiency due to the two effects (already discussed) cancelling each other [22,61]. For example one study reported that the two salts, sodium chloride and sodium sulphate, were found not to influence the HF-LPME of several NSAIDs in wastewater [64]. However, its impact is limited at environmentally relevant salt concentrations with some multivariate-based analysis studies observing that ionic strength was not a critical parameter and went on to do their experiments without salt addition [16,39]. In addition, further evidence suggests that the addition of salt into the donor phase is not beneficial for shorter extraction times, but may be useful in longer extractions [71].

### 3.7. Matrix Effects

When compared to SPE, HF-LPME has been shown to be less affected by highly particulate samples, displaying only minor matrix effects [9]. As a result, the influence of humic acids in HF-LPME has been regarded as a minor factor which has not been investigated in numerous studies. In one case, humic acid did not influence the extraction efficiency of two estrogens (17-β-estradiol and estrone), with its increasing concentration from 0 to 25 mg L^−1^ only affecting diethylstilbestrol recovery [15]. Although slightly affected by the presence of humic acid in the sample solution, the diethylstilbestrol recovery remained above 80% [15]. In a different study, the presence of humic acid in aqueous solutions at the concentration range of 0 to 25 µg mL^−1^ did not influence the extraction efficiency of sulphonamides [71]. These results were translated to no significant matrix effect observed during the extraction of sulphonamides [71].

## 4. Performance of HF-LPME in the Analysis of Pharmaceuticals in Water

HF-LPME produces cleaner extracts and results in detection of lower concentrations of analytes [62]. In a comparative study for the analysis of NSAIDs using liquid chromatography equipped with both diode array and fluorescence detectors (LC-DAD-FLD), HF-LPME led to higher enrichment factors of 261–301 when compared to 89–176 achieved using SPE in the same study [62]. Some researchers have compared their proposed HF-LPME based analytical methods with the existing procedures reported in the literature [12,44,50]. In this context, an HF-LPME based analytical method reported by Asadi et al., 2016 for the analysis of naproxen and nabumetone yielded lower detection limits when compared to solid-phase microextraction methods reported in the literature [50]. This was attributed to higher enrichment factors [50]. A recent article reported a detection limit of 2.8 µg L^−1^ for carbamazepine which is lower than the reported range of 6–80 µg L^−1^ achieved using the methods that are based on stir bar sorptive extraction, microextraction by packed sorbent, and dispersive liquid-liquid microextraction [12]. Similarly, Shariati et al., 2009 reported that their proposed HF-LPME based analytical method for tetracycline antibiotics had a higher sensitivity and better precision than other procedures reported in the literature utilizing the same analytical technique but with a different sample preparation method [44].

The detection limits achieved using the HF-LPME technique for analysis of pharmaceuticals in water are summarized in Table 1. The ability of HF-LPME to pre-concentrate pharmaceuticals in water is well demonstrated in Table 1, where the detection limits achieved using less sensitive analytical instrumentation such as LC-FLD, LC-DAD, and gas chromatography with flame ionization detector (GC-FID) are in the low ng L^−1^ levels (typically <50 ng L^−1^) [50,54,56,58,62,69]. As such, much lower detection limits in low pg L^−1^ have been reported when using LC-MS instrumentation [18,41]. This could be further credited to high enrichment factors of up to 27,000 achieved by Ho et al., (2007), which led to detection limits in the range of 6–31 pg L^−1^ for antidepressants [41]. This means, the HF-LPME is suitable for the treatment of environmental samples as it is capable of selectively isolating and pre-concentrating a wide range of analytes with similar properties.

## 5. Improvements of HF-LPME Based Methods for Pharmaceutical Analysis in Water

Despite the documented advantages of HF-LPME over other extraction techniques [26], the HF-LPME technique still has its own drawbacks. The method is relatively slow with some studies on analysis of pharmaceuticals reporting between 2–8 h of extraction time [9,15,18,56,58]. In addition, the set-up of the HF-LPME method is flexible which limits potential for standardized equipment that can be commercialized [26]. The method is therefore not considered user-friendly and self-made set-ups in the laboratory may affect reproducibility. For example, the volume of acceptor phase recovered from the same length of fibre is always different, yet volume is an important parameter in calculation of analyte recovery in the acceptor phase. In addition, Quintana et al., 2004 observed low inter- and intra-day repeatability recording relative standard deviation values of up to 30 and 32% respectively for analysis of pharmaceuticals in wastewater [11]. Other drawbacks include formation of air bubbles and accumulation of hydrophobic substances at the aqueous phase–organic phase interfaces which tend to reduce transfer rate [77,78]. To counteract these drawbacks, various advances have been made on the conventional HF-LPME set-up including applying a DC current (electro-membrane extraction-EME) and the development of solvent bar micro-extraction (SBME) to enhance transfer and reduce extraction times as well as replace the acidified acceptor phases with green solvents (ionic liquids and deep eutectic solvents) [30,32,79,80,81]. These and other improvements are discussed in the following sub-sections.

### 5.1. Advances in Supported Liquid Membrane

Various studies have been undertaken to counteract issues associated with the stability of the SLM in the HF-LPME approach. Strip dispersion and feed dispersion methods have been evaluated as ways of combating the membrane instability and improving extraction efficiency of targeted analytes. The inclusion of a disperser in the strip solution reduced the resistance on the boundary of the membrane, in turn creating greater mass transfer and higher enrichment [82,83]. With the strip dispersion method, the extraction of cephalexin from the donor solution was circa 99% and the recovered amount from the strip solution employing aliquat 336 as extractant was 98% [82]. The same research group [82] also demonstrated that the SLM-feed dispersion (SLM-FD) was found to yield 1.7 times higher extraction efficiencies for cephalexin than SLM-strip dispersion due to the constant supply of the membrane with organic droplets. Another invention to address the membrane instability in the traditional SLM was the incorporation of ionic liquids, whose chemical composition consists of an organic cation and inorganic or organic anion with melting points below 100 °C, as part of the liquid membranes [45,84]. Very stable ionic liquid immobilized polymeric support (SILM) can be prepared through proper selection of ion pairs [85,86]. Thus, the inclusion of ionic liquids in supported ionic liquid membranes imparts high thermal stability and low vapour pressure owing to their viscosity and high surface tension, hence preventing the loss of organic phase to the aqueous phase [84]. Recently, the exploration of deep eutectic solvents in SLM was reported [87]. Over 99% recovery was found with the integrated SLM-SD process involving crystallization for the recovery and separation of amlodipine chiral enantiomers [88].

### 5.2. Application of Green Solvents in the Extraction Process

HF-LPME is viewed as a greener sample preparation technique when compared to other methods that are based on SPE and LLE. This is mainly because HF-LPME methods use micro-volumes of solvents and reduced sample sizes. One of the twelve principles of green analytical chemistry is based on the elimination of toxic reagents [70]. In greening the HF-LPME based methods for pharmaceuticals, various authors have proposed the replacement of hazardous organic solvents used in the extraction process with greener chemicals such as ionic liquids [71] and deep eutectic solvents [75,76]. Applications of ionic liquids in HF-LPME of pharmaceuticals in environmental water samples is currently limited. In this context, only the ionic liquid, 1-octyl-3-methylimidazolium hexafluorophosphate as an SLM in the presence of tri-*n*-octylphosphine oxide as a carrier has been reported for extraction of 17-β-estradiol and estrone in river water [15] and 5 sulfonamides in river and wastewater [71].

However, ionic liquids are well reported in HF-LPME of other analytes as green solvents that can be used as acceptor solutions for pharmaceuticals especially in bioanalysis [89,90,91]. The application of ionic liquids for the extraction of pollutants is dependent on their properties. Such properties which are described in our previous work include low vapor pressure, high thermal stability, miscibility with a wide range of organic solvents, good extractability for many different organic, inorganic, and organometallic materials, high viscosity as well as high ionic conductivity [92]. The drawback which limits the extensive application of ionic liquids is their high cost which has led to the introduction of the much cheaper deep eutectic solvents.

Deep eutectic solvents are actually a subclass of ionic liquids which are easily synthesized with low cost raw materials [93]. In simple terms, deep eutectic solvents are mixtures of a hydrogen bond donor and a hydrogen bond acceptor that form liquids due to a large depression of the melting point [75]. The interests for the application of deep eutectic solvents in analytical chemistry procedures is related to their favourable physical properties which include low volatility, good thermal stability, high conductivity, tunable miscibility, biodegradability, biocompatibility, non-toxicity, and non-flammability [94]. Seidi et al., (2019) utilized a deep eutectic solvent prepared from ethylene glycol (hydrogen bond donor) and choline chloride (hydrogen bond acceptor) as an acceptor phase in HF-LPME of raloxifene and ethinylestradiol from pharmaceutical wastewater [75]. Upon optimization (optimum parameters in Table 1), their LC-UV based analytical method gave detection limits of 5 and 10 µg L^−1^ for raloxifene and ethinylestradiol, respectively [75]. In the HF-LPME of antiarrhythmic agents from pharmaceutical wastewater, a deep eutectic solvent prepared from choline chloride and 1-phenylethanol was used as the extraction solvent without the utilization of any carrier chemical [76].

In summary, there are more reports in the literature where both deep eutectic solvents [91,95,96] and ionic liquids [89] have been applied as acceptor phase and supported liquid membranes in the HF-LPME technique of a wide range of analytes in various sample matrices. However, there are still very few articles reporting the applications of these chemicals in HF-LPME of pharmaceuticals from water samples [75,76]. The applications of ionic and deep eutectic solvents as SLMs for HF-LPME is limited by their high solubility in aqueous solutions [21]. This is an area that is likely to be more explored in future since the HF-LPME is a promising tool for efficient extraction and pre-concentration of pharmaceuticals in environmental waters, thereby resulting in selective and sensitive analytical methods.

### 5.3. Inclusion of Solid Sorbents

LPME is a miniaturized version of LLE [97]. Its applications and performance has been enhanced by incorporating solid particles in the acceptor phase, thereby, altering the extraction process by introducing the elements of SPE [97,98,99]. In the extraction set-up known as hollow fibre solid-liquid phase microextraction, the acceptor phase is prepared by dispersing the adsorbent which acts as the analyte trapper into the organic solvent which is then transferred into the lumen of the hollow fibre. Thereafter, the normal HF-LPME process is followed with the inclusion of desorption step prior to chromatographic analysis of the extracted compounds. The choice of the adsorbent influences the selectivity of the analytical method. The adsorbents can also impact the sensitivity of the analytical method as the application of materials with large number of active adsorption sites and porosity could lead to the attainment of high pre-concentration factors. In the analysis of β-blockers, functionalized multi-walled carbon nanotubes in 1-octanol were used as the acceptor phase attaining detection limits ranging from 1 to 15 µg L^−1^ when using LC-FLD for analysis of tap water, clinical wastewater, and industrial wastewater [97]. The same acceptor solution (multi-walled carbon nanotubes in 1-octanol) has been utilized for piroxicam and diclofenac in an LC-DAD analytical method that yielded quantitation limits of 12.0 and 3.6 µg L^−1^, respectively [99]. For ibuprofen and naproxen (NSAIDs), the detection limits for clinical wastewater analysis were 2.95 and 1.51 µg L^−1^, respectively, when using LC-UV after performing extraction utilizing hyperbranched polyglycerol/graphene oxide nanocomposite in 1-octanol as the acceptor phase [98]. This innovation is likely to produce cleaner extracts during the pharmaceutical analysis with less chromatographic errors when using highly selective adsorbents such as molecularly imprinted polymers (MIPs). The application of MIPs for this purpose has already been reported for the extraction of a few pharmaceuticals which include diclofenac [100] and fluoroquinolone antibiotics in environmental waters as well as urine samples [101]. However, the synthesis of MIP is conducted in situ, with Barahona et al., (2019) presenting the MIP synthesis conducted inside the pores of hollow polypropylene fibres followed by application in the extraction of fluoroquinolone antibiotics in environmental waters and urine samples [101].

### 5.4. Automated and Continuous Flow HF-LPME

The twelve principles of green analytical chemistry include one that encourages the applications of automated and miniaturized methods [70]. HF-LPME is already a miniaturized version of LLE. Automated HF-LPME has already been discussed in the literature for several environmental pollutants which include pesticides [102], chlororobenzenes [103], and perfluorinated compounds [104]. In the context of pharmaceutical analysis in water, automated HF-LPME has been reported for the determination of two hormonal drugs, megestrol acetate and levonorgestrel in water and urine samples [19]. The extraction procedure utilized methanol and *n*-dodecane as acceptor phase and supported liquid membrane, respectively, while LC-UV was the analytical technique yielding a detection limit of 250 ng L^−1^ (Table 1) for both compounds [19]. Although the automation of experimental procedure is much desired from the green chemistry perspective, its set-up is likely to introduce a financial burden in the analysis as it has to be sourced at a price. This drawback could be a reason for limited applications of automated HF-LPME of pharmaceuticals in water.

**Figure 3:**
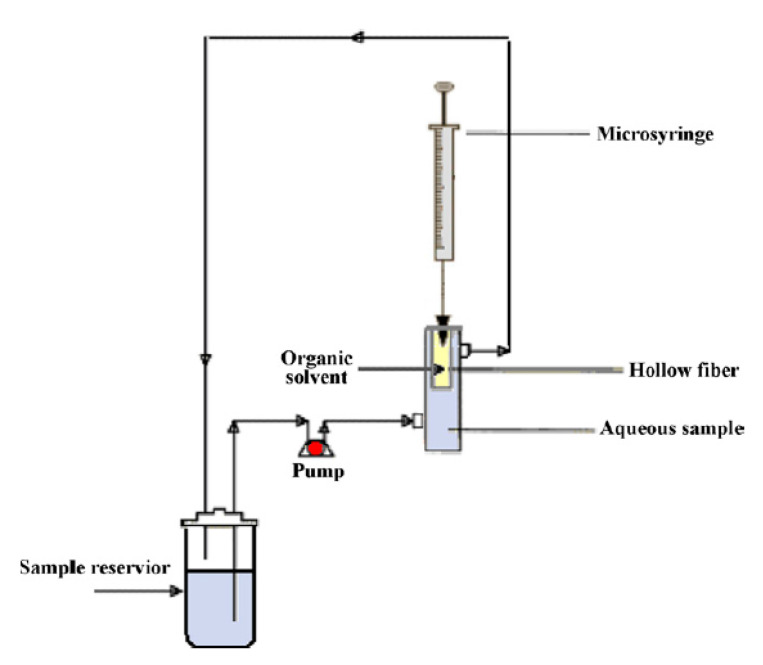
Schematic diagram of the experimental setup of the continuous hollow fibre-liquid-phase microextraction [54].

Continuous flow HF-LPME was reported over a decade ago for the isolation and pre-concentration of NSAIDs, antibiotics, and oestrogens from aqueous samples prior to GC-FID [54] and HPLC-DAD/FLD [60,62]. This version of HF-LPME extracts analytes from continuous flowing solutions through the SLM into acceptor solutions. The schematic diagram of the basic extraction system is given in Figure 3. This approach can be used for on-site measurements leading to the determination of concentration peaks or time weighted average concentrations [62]. The application of this technique with GC-FID has yielded very low detection limits ranging from 1 to 2 ng L^−1^ for selected NSAIDs (ibuprofen, naproxen, and ketoprofen) in water samples [54].

## 6. Environmental Monitoring of Pharmaceuticals Using HF-LPME

Applications of HF-LPME in analysis of pharmaceuticals has been mentioned briefly recently in some reviews including a review of the extraction methods for pharmaceuticals in aqueous samples [105], the analysis of pharmaceuticals in biological samples [43,106,107], and a further one on environmental and bioanalytical applications of HF-LPME in 2008 [108]. In the current review, we give an in-depth review of its applications specifically in the analysis of pharmaceuticals in environmental aqueous samples. Table 1 summarizes the modes, the optimum conditions, and the pharmaceuticals that have been analysed using this technique. The HF-LPME technique has been utilized as an effective alternative for selectively isolation and pre-concentration of pharmaceuticals in aqueous samples. It has been documented in numerous studies that pharmaceutical levels in environmental waters are low hence sensitive analytical tools are still crucial for environmental monitoring [3,109]. The most applicable analytical technique is liquid chromatography due to non-volatility of most pharmaceuticals. Various detection systems have been utilized for environmental monitoring of pharmaceuticals (Table 1). Some analytical methods were able to detect pharmaceuticals in environmental samples at low ng L^−1^ to µg L^−1^ levels. Based on Table 1, HF-LPME based methods are generally widely applied in the analysis of wastewater samples rather than other water matrices such as surface water and seawater.

## 7. Conclusions

Both the two- and three-phase configurations of HF-LPME have been widely applied for the isolation and pre-concentration of pharmaceuticals from various water matrices. HF-LPME has been proven as an ideal sample preparation technique that provides cleaner extracts and high enrichment factors which impact the sensitivity of analytical methods. In addition, the extractable compounds are neutral in the sample solution which promotes the selectivity of the analytical methods. Selectivity can also be enhanced by the introduction of selective sorbents such as MIPs inside the lumen of the hollow fibre which act as the acceptor phase. Furthermore, the HF-LPME is a green analyte extraction technique which uses low volumes of organic solvents. The latest research has shown that even those small volumes of traditional organic solvents can be replaced by using environmentally friendly chemicals such as ionic liquids and deep eutectic solvents. However, it was observed that the automation of HF-LPME is still limited due to unavailability of commercial equipment. The application of HF-LPME precludes any possibility of analyte/sample carryover as the hollow fibre is cheap enough to be discarded or disposed after single use. Overall, the HF-LPME is a simple process. Its flexible set-up allows for advanced modifications in various ways including automation, miniaturization, and the potential substitution of environmentally unfriendly organics with green solvents. In this regard, the HF-LPME technique remains a viable alternative in the analysis of pharmaceuticals (and other organic and inorganic substances) for environmental as well as biological samples.

## Figures and Tables

**Table 1:** Performance of hollow fibre-liquid phase microextraction in the analysis of pharmaceuticals in water samples.

Pharmaceuticals	Matrix, Volume and Its pH	Supported Liquid Membrane + Carrier Molecule Composition	Acceptor Phase, Its Volume and pH	Stirring Rate (rpm), Extraction Time (min)	Analytical Technique	Detection Limits (ng L^−1^)	Reference
Naproxen and nabumetone	9 mL WWTP influent and tap water, pH 3	-	14 μL of 1-undecanol	600, 20	LC-FLD	1.3–2.9	[50]
Ibuprofen, naproxen, and ketoprofen	20 mL tap water, wastewater and surface water, pH 3.5	1-octanol	4 μL of octanol	* 1 mL min^−1^, 20	GC-FID	1–2	[54]
Amitriptyline, clomipramine, doxepin, mianserin and nortriptyline	100 mL wastewater, pH 11.8	di-n-hexyl ether	20 µL of 10 mM formic acid	800, 60	LC-MS	0.006–0.031	[41]
Salbutamol and terbutaline	11 mL environmental water (pH 11)	dihexyl ether + 20% (*w*/*v*) Aliquat 336	24 µL 1M NaBr	50, 60	LC-DAD	500–2500	[47]
17-β-ethynylestradiol, 17-β-estradiol, estrone	100 mL tap and sewage water	di-n-hexyl ether + 10% (*w*/*v*) TOPO	10 µL of n-undecane	1 100, 2	GC-MS	1.6–10	[34]
Ketoprofen, naproxen, diclofenac and ibupprofen	1 L WWTP effluent, pH 1.5–2	di-n-hexyl ether	aqueous solution at pH 9.5	** 30 mL min^−1^, 45	LC-DAD-FLD	10–50	[62]
4 NSAIDs and 8 of their metabolites	50 mL wastewater, pH 2	di-n-hexyl ether + 5% (*w*/*v*) TOPO	10 µL of 0.1 M ammonium carbonate, pH 9	660, 5 h	LC-MS	7.1–89.3 µg L^−1^	[9]
Carbamazepine	12 mL wastewater, well and river waters, pH 8.9	octanol	25 µL octanol	400, 48.5	LC-DAD	2800	[12]
6 β-blockers	55 mL wastewater, pH 11.5	heptanol	25 µL heptanol	800, 60	LC-UV	80–500	[13]
3 antiretroviral drugs	10 mL surface and wastewater, pH 4	dihexyl ether + 5%, (*w*/*w*) DEHPA	22.6 μL of 0.4 mM HCl	1000, 60	LC-MS	9–160	[16]
4 NSAIDs	6 mL wastewater and surface water, pH 3	dihexyl ether + 5%, (*w*/*w*) di-(2-ethylhexyl) phosphoric acid	22.6 µL of aqueous solution (pH 10)	900, 60	LC-MS	0.05–0.35	[39]
7 NSAIDs	50 mL wastewater, pH 2	Dihexyl ether	30 µL of aqueous solution (pH 12)	300, 20	CE	205–860	[64]
Ibuprofen, naproxen, and ketoprofen	2.5 mL pure water containing 250 µL 0.1 M HCl	Dihexyl ether	25 µL of 10 mM NaOH	400, 45	CE	5000	[17]
Ibuprofen and clofibric acid	4 mL of 0.1 M HCl wastewater solution	1-octanol	100 µL 0.01 M NaOH	700, 40	LC-UV	15–100	[38]
5 sulfonamides	4 mL river and wastewater (pH 4.5)	ionic liquid + 14% (*w*/*v*) TOPO	25 µL aqueous solution (pH 13)	300, 8 h	LC-DAD	100–400	[71]
Ketoprofen, naproxen, and clofibric acid	10 mL of 0.01M HCl wastewater solution	1-octanol	5 µL of 0.5M NaOH	73 rad s^−1^, 60	LC-UV	30–300	[72]
4 sulfonamides and their main metabolites	50 mL wastewater, river and tap water, pH 4	1-octanol	50 µL aqueous solution, pH 12	300, 6 h	LC-DAD-FLD	0.3–33	[58]
Sulphonamides	Water samples (pH 6)	5% TOPO in hexylamine	0.4 M H_2_SO_4_	Continuous flow at 0.3 mL min^−1^ for 60 min	LC-DAD	<20 µg L^−1^	[60]
Steroids	Water samples (pH 6)	n-undecane/di-n-hexyl ether (1:1 *v*/*v*) + 5% (*w*/*v*) TOPO	0.4 M H_2_SO_4_	Continuous flow at 0.1 mL min^−1^ for 60 min	LC-DAD	<2.4	[60]
Salicylic acid, diclofenac, and ibuprofen	50 mL wastewater, pH 2	Dihexyl ether	50 µL aqueous solution, pH 12.5	300, 15	LC-MS	20–300	[55]
8 fluoroquinolones	50 mL wastewater, river water and tap water, pH 7	1-octanol	50 µL aqueous solution, pH 12	300, 5.5 h	LC-DAD-FLD	0.3–16	[56]
9 NSAIDs	22 mL wastewater, pH 2	1-octanol	20 µL of 10 mM ammonium carbonate	500, 45	LC-MS	0.5–42	[11]
Tetracycline, oxytetracycline, and doxycycline	11 mL tap water, pH 9	1-octanol + 10% (*w*/*v*) aliquat-336	24 µL of 0.1 M H_3_PO_4_ and 1.0 M NaCl, pH 1.6	900, 35	LC-UV/Vis	500–1000	[44]
Megestrol acetate and levonorgestrel	20 mL water, pH not adjusted	n-dodecane	25 µL methanol	1000, 40	LC-UV/Vis	250	[19]
5 selective serotonin reuptake inhibitors and 4 of their metabolites	1.1 L seawater and wastewater, pH 11.8	Dihexyl ether	20 µL aqueous solution, pH 2	800, 2 h	LC-MS	0.017–0.618	[18]
4 fluoroquinolone antibiotics	10 mL surface water (pH 6)	di-n-hexyl ether + 20% (*w*/*w*) DEHPA	56.5 µL of 0.1 M HCl	200, 2 h	LC-DAD	10–20	[37]
carbamazepine ibuprofen, phenazone, 17-α-ethinylestradiol	5 mL water sample (pH 2)	1-octanol	17 µL 1-octanol	1 000, 60	GC-MS	20–40	[73]
diethylstilbestrol, dienestrol, and hexestrol (oestrogens)	10 mL wastewater (pH 1.5)	1-octanol	10 µL of 0.5 M NaOH	1 200, 40	LC-UV/Vis	250–500	[59]
8 sulfonamides	8 mL wastewater, pH 3.5	1-octanol	30 µL NaOH, pH 12.5	600, 75	LC-FLD	3.1–11.2	[69]
Salicylic acid, para-aminosalicylic acid and acetylsalicylic acid	10 mL sea and river water, pH 3	1-octanol	15 µL purified water pH 6.2	1000, 45	LC-UV/Vis	600–1200	[74]
4 NSAIDs	5 mL purified water, tap water, pH 1.5	1-octanol	15 µL 1-octanol	300, 20	LC-MS	500–1250	[14]
17-β-estradiol, estrone and diethylstilbestrol	50 mL river water, pH 2	Ionic liquid	2.5 µL ionic liquid	200, 8 h	LC-UV/Vis	50–100	[15]
11 antibiotics	20 mL river water, pH 8	dihexyl ether + 20% (*w*/*v*) aliquat-336	20 µL acetic acid, pH 4	200, 60	LC-MS	10–250	[68]
Raloxifene and ethinylestradiol	17 mL pharmaceutical wastewater, pH 11	1-octanol + 0.04 g mL^−1^ CTAB	20 µL deep eutectic solvent	700, 42	LC-UV	5000–10,000	[75]
4 anti-arrhythmic agents	10 mL pharmaceutical wastewater, pH 12.3	ChCl:Ph-ETOH	40 µL aqueous solution, pH 2.5	1100, 40	LC-UV	300–800	[76]
27 emerging contaminants included pharmaceuticals	1000 mL river water, pH 7	1-octanol	60 µL of 1-octanol	100, 30	LC-MS	1.09–98.15	[53]

LC: Liquid chromatography; GC: Gas chromatography; MS: mass spectrometry; FLD: Fluorescence detector; FID: Flame ionization detector; UV/Vis: Ultraviolet/visible detector; DAD: Diode array detector; * sample circulation speed; ** continuous flow method. CE: Capillary electrophoresis; Ionic liquid: 1-Octyl-3-methylimidazolium hexafluorophosphate; CTAB: *N*-Cetyl-*N*,*N*,*N*-trimethylammonium bromide; TOPO: tri-n-octylphosphine oxide, DEHPA: di-(2-ethylhexyl) phosphoric acid; WWTP: wastewater treatment plant; NSAIDs: Non-steroidal anti-inflammatory drugs; NaBr: sodium bromide; HCl: Hydrochloric acid. - Information is not provided.
